# Supplementary material for: Genetic immune escape landscape in primary and metastatic cancer
Source: Nat Genet. 2023 May 10;55(5):820–31. doi: 10.1038/s41588-023-01367-1 (PMC10181939; doi:10.1038/s41588-023-01367-1)
Supplement: Supplementary file 1 — Supplementary Tables 1 and 2 and Data 1–4 captions, and Supplementary Notes 1–3. [file 41588_2023_1367_MOESM1_ESM.pdf]

nature genetics

Article https://doi.org/10.1038/s41588-023-01367-1

Genetic immune escape landscape in primary and metastatic cancer

In the format provided by the authors and unedited

Supplementary Information

Supplementary Tables

Supplementary Table 1. Pathways and genes involved in genetic immune escape.

Supplementary Table 2. Datasets metadata and cancer type representation.

Supplementary Data

Supplementary Data 1. *HLA-I* typing for Hartwig and PCAWG patients and validation of LILAC.

Supplementary Data 2. Sample specific GIE annotation and cohort-wise GIE frequency.

Supplementary Data 3. Positive selection in *HLA-I* and non *HLA-I* genes.

Supplementary Data 4. Tumor genomics and clinical features and GIE association.

Supplementary Notes

Supplementary Note 1. LILAC.

Supplementary Note 2. Neoepitope prioritization pipeline.

Supplementary Note 3. GIE and tumor genomic features.

Supplementary Information 1
Supplementary Tables 1
Supplementary Data 1
Supplementary Notes 1

Supplementary Note 1:LILAC 3
Overview 4
HLA-I typing algorithm 4
Elimination phase 5
1. Nucleotide matrix 5
2. Amino acid matrix 6
3. Phased haplotypes 6
4. Recover common alleles 7
5. Detect HLA-Y presence 7
6. Test for 2 digit types with unique evidence 7
7. Remove incomplete alleles with insufficient unique evidence 7
Evidence phase 7
Tumor and RNA status of alleles 9
Tumor allele specific copy number 9
Somatic variant assignment to alleles 9
RNA expression of alleles 9
Benchmark 10
Germline-tumor agreement comparison 10
Crosswise tools HLA-I haplotype comparison 10
HLA-I typing performance using Platinum and Yoruba family trios 10
HLA-I typing agreement with the TRACERx 100 lung cohort 11
Copy number estimation of HLA-I compared to other genes 11
LILAC's LOH of HLA-I agreement with LOHHLA in TRACERx cohort 11
Experimental validation by high-to allelic resolution HLA typing 12
Application to Hartwig and PCAWG datasets 12
Usage 12

Supplementary Note 2: Neoantigen pipeline 14
Overview 14
Workflow 14
1. Identification of candidate neoepitopes 14
2. Calculation of allele specific binding affinity and presentation scores 16
2.1 pHLA algorithm description 16
2.1.1. Conventions regarding positions, peptide lengths and HLA allele binding motifs 16
Position Independence 16
Peptide length mappings 17
Flanking sequences 17
Binding Motifs 17

Binding Motif similarity 18
2.1.2. Training dataset 18
2.1.3 Construction of Position Weight Matrix (PWM) 19
2.1.4. Scoring and ranking per allele per peptide length 20
2.1.5. Relative presentation likelihood and ranking per allele (pan peptide length) 20
3. Expression adjusted presentation likelihood algorithm 22
3.1. Training data 22
3.2. TPM adjusted likelihood rank 22
Neoepitope identification in Hartwig and PCAWG dataset 23
Neoepitope clonality 23
Neo pipeline validation 23
1. Neo performance compared to MHCFlurry2.0 24
2. Neo ranking of random peptides. 26
3. Neo robustness by performing an allele leave-one-out experiment. 27
4. Neo expression adjusted model performance 30
Usage 31
Supplementary Note 3: GIE and tumor genomic features 32
Tumor genomic features and GIE association 32
Background simulation of GIE alterations 32
Tumor mutation burden, neoepitope load and SV load 33
Mutational signatures 33
MMR and HR deficiency 35
DNA viral insertion and Whole Genome Duplication 35
Immune infiltration deconvolution 35
HLA-I supertypes 35
HLA-I divergence 36
Pre-biopsy treatment exposure 36
Driver alterations 36
References 37

Supplementary Note 1:LILAC

Overview

LILAC is a WGS framework to determine the HLA class I types for the germline of each patient as well as determining the status of each of those alleles in the tumor including complete loss of one or more alleles, allele specific somatic mutations and allelic imbalance.

LILAC provides several conceptual and practical improvements over the numerous available tools for HLA-I typing: i) increased accuracy for WGS samples with coverage between 30-100X, particularly remarkable for rare alleles, ii) integrated analysis of paired tumor-normal sample data to call allele-specific copy number and assignment of somatic variants to specific alleles, iii) detection of novel germline variants and/or alleles (including indels) via analysis of unmatched fragments iv) full report of quality control metrics and number of fragments assigned to each HLA-I allele and, finally, v) identification of HLA-Y presence (a pseudogene with high similarity to HLA-A present in up to 20% of the population but is not present in the reference genome).

LILAC relies on the somatic point mutations and copy number estimations to estimate the tumor HLA-I status. LILAC also works for whole-exome sequencing. Lastly, LILAC supports GRCH37, hg19 and hg38 (with no alt) reference genomes (Fig. 1b from main text). LILAC is freely avaliable at https://github.com/hartwigmedical/hmftools/tree/master/lilac.

HLA-I typing algorithm

The starting point for the LILAC algorithm is the complete set of possible 4 digit alleles and all the fragments aligned to HLA-A, HLA-B and HLA-C. Where multiple 6 digit or 8 digit types are present in the IMGT/HLA database[1], LILAC uses the numerically lowest type for all calculations. Note that 2 HLA-A alleles {A*31:135, A*33:191} have been removed from the database due to it frequently being found as a low level artifact (likely due to the high similarity to closely related genes and pseudogenes such as HLA-H).

LILAC algorithm begins with collecting all fragments which are not duplicates and have:

- At least one read with an alignment overlapping a coding base of HLA-A, HLA-B or HLA-C; and
- all alignments within 1000 bases of a HLA coding region; and
- a mapping quality of at least 1

The algorithm then has 2 main phases to determine the germline alleles: an *elimination* phase which aims to remove allele candidates that are clearly not present and an *evidence* phase where LILAC considers all possible sets of 6 alleles amongst the remaining candidates and chooses the solution that best explains the fragments observed.

After the germline alleles are determined, LILAC determines the tumor copy number and any somatic mutations in each allele. Note that if more than 300 bases of the HLA-A,HLA-B and HLA-C coding regions have less than 10 coverage, then LILAC will fail with errors and will not try to fit the sample.

Elimination phase

The elimination phase is primarily an optimization. Its goal is simply to reduce the number of possible alleles from ~14k present in the IMGT/HLA database to a manageable number such that the evidence phase can run efficiently. The principle in the elimination phase is to remove any allele that does not have at least a certain minimal coverage of each of it's amino acids and bases. To mitigate the chance of inadvertently eliminating a true allele, common alleles may be recovered at the end of the elimination phase if they have sufficient unique support, but are then penalized in the subsequent evidence phase relative to other candidate alleles.

The steps in the elimination phase are:

1. Nucleotide matrix

At each coding position, create a matrix of (high quality) nucleotide count and determine all bases which are heterozygous across all 6 alleles.

We do this 3 separate times for HLA-A, B and C. Each time we consider fragments from any of the alignment records that have similar exon boundaries to the type in question. For instance, fragments from the earlier exons which have identical boundaries across all 3 genes will be used to construct the A, B and C matrices, but fragments from the later exons may only contribute to A and B or perhaps only C. The counts of supporting fragments are then aggregated at each position to construct the nucleotide matrix.

Fragments with in-frame indels are only included if the indel matches an existing hla type allowing for realignment. Fragments with out-of-frame indels are always excluded (note for the special case of C*04:09N, a relatively common allele with out of frame indel, it is explicitly rescued at a later stage

During the elimination phase, nucleotide candidates are filtered to include only those with at least max(1,0.000375 * FragmentCount) high quality (base qual > min(30,medianBaseQuality)) fragment and at least max(2,0.00075 * FragmentCount) fragments overall support. Subsequently, base quality is not considered. Sites with more than 1 nucleotide candidate are deemed heterozygous and sites with only 1 are considered to be homozygous across all 6 alleles.

Any alleles with bases that do not match both the heterozygous and homozygous locations of the nucleotide matrix are eliminated.

2. Amino acid matrix

Similarly to the nucleotide matrix, LILAC also constructs a matrix of amino acid candidates. Again, amino acid candidates are filtered to those where at least max(1,0.000375 * FragmentCount) fragments support with high base quality (all 3 nucleotides) and at least max(2,0.00075 * FragmentCount) fragments over all. The codon matrix can include inframe insertions and deletions where these match at least one known allele (base quality is not considered).

Exon boundary 'enrichment' is applied for all shared amino acids across all 3 genes (amino acids index < 298). This enriches any fragment with nucleotides on one side of an exon boundary with any homozygous nucleotides from the other side so that an amino acid is able to be constructed.

Similarly to the nucleotide matrix, any alleles with an amino acid or inframe indel that do not match the amino acid matrix are eliminated.

3. Phased haplotypes

In this step we phase the heterozygous amino acid locations and eliminate any alleles that are not supported by phased locations with sufficient overall shared coverage.

First, we find phased evidence of each consecutive pair of heterozygous codons and record the haplotypes of all fragments containing both codons. This is performed separately for each of HLA-A, HLA-B and HLA-C to account for differences in exon boundaries. Fragments which overlap amino acid 338 onwards will only be assignable to a subset of the alleles, since the exon boundaries differ after this amino acid. The points are only phased if the total coverage is at least 7 fragments per allele [minFragmentsPerAllele] included in the subset at that location (ie. between 14 and 42 fragments with shared coverage depending on the amino acid location). A phased haplotype with only 1 supporting fragment will be removed if the total fragments supporting the pair is 40 or more [minFragmentsToRemoveSingle] (assumed to be a sequencing error).

We then iteratively choose the phased haplotype with the most support and perform the following routine:

- Find other phased evidence that overlaps it.
- Find the minimum number of codon locations required to uniquely identify each phased evidence, eg, if the left evidence has haplotypes [SP, ST] you only need the last codon but if the right evidence has haplotypes [PD, TD, TS] you would need both codon locations.
- Find evidence of fragments that contain all the required codons. As with the paired evidence, there must be at least at least 7 fragments per allele supporting the pair [minFragmentsPerAllele] and a haplotype with only 1 supporting fragment will be removed if the total fragments supporting the pair is 40 or more [minFragmentsToRemoveSingle].
- Check that the new overlapping evidence is consistent with the existing evidence.
- Merge the new evidence with the existing paired evidence.

- Replace the two pieces of used evidence with the new merged evidence.

Once complete, we can eliminate any alleles that do not match the phased evidence.

4. Recover common alleles

As a fail safe for phasing, any ‘common alleles’ with more than 0.1% population frequency are recovered. The frequencies of alleles are specified in a resource file and are derived from the Hartwig cohort.

Additionally, C*04:09N (the most common HLA allele with a frameshift variant) specifically is also rescued if the out of frame indel 6:31237115 CN>C is present.

5. Detect HLA-Y presence

HLA-Y is a pseudogene that is highly similar to HLA-A and is not present in the human ref genome but is found in approximately 17% of the Hartwig cohort. The presence of HLA-Y can cause confusion in typing particularly in determining the HLA-A types. To detect HLA-Y, LILAC counts the number of fragments that can be assigned uniquely to one of the 3 known HLA-Y alleles and no other candidate alleles. If at least 1% of fragments align uniquely to HLA-Y then HLA-Y is considered to be present in the sample. If HLA-Y is found to be present ANY fragment which matches exactly to a HLA-Y allele (uniquely or shared with other alleles) are excluded from further analysis to prevent confusion with highly similar HLA-A alleles.

6. Test for 2 digit types with unique evidence

To further reduce the number of candidate alleles, If any 2-digit types are sufficiently unique (i.e. uniquely supported by at least 2% of fragments, they are required to contain at least one 4 digit type belonging to that 2 digit type in the evidence phase. If two 2 digit types from the same gene are found to be sufficiently unique all other alleles are discarded at this point. If more than two groups are found to be unique the 2 with the highest evidence are supported Any recovered alleles are also discarded at this point unless the 2 digit group has at least one fragment of unique support.

7. Remove incomplete alleles with insufficient unique evidence

Many alleles in the IMGT database are incomplete (ie contain ‘*’ characters), all of which are rare in population frequency. To prevent spurious matches to these wildcard containing alleles in the evidence phase, we eliminate unlikely candidates. Wildcard containing alleles are eliminated unless they contain at least 2 fragments support for the non wildcard sequence which do not support any remaining candidate allele with a complete sequence defined.

Evidence phase

In the evidence phase, LILAC evaluates all possible ‘complexes’ (ie. combinations) of remaining alleles that satisfy the following conditions

- There must be either 1 (homozygous) or 2 (heterozygous) alleles belonging to each gene
- At least 1 allele must match each uniquely supported 2 digit type

For each complex, LILAC counts the number of fragments that can be aligned exactly to at least one allele in the complex at all heterozygous locations. If a fragment has an amino acid which does not match ANY of the amino acid candidates at a heterozygous location, but has at least 1 nucleotide with base qual<min(medianBaseQuality,30), then the amino acid is deemed to match all amino acid candidates For exon boundaries only exact nucleotide matches are permitted. Any fragments that can be aligned to 2 or more alleles are apportioned equally between the alleles and counted as shared fragments.

Since many allele definitions have undetermined ('wildcard') sequences, particularly in exon 1 and exons 4-8, these require special treatment so that these wildcard alleles are neither unfairly favored or discriminated against in the fitting. To achieve this balance, fragments which don't match an exact sequence in any candidate allele are dropped altogether from consideration such that random sequencing errors or other artifacts overlapping wildcard regions cannot contribute to the complex count for wildcard containing alleles, but any remaining fragments are considered to match an allele if they match all non wildcard sequences (ie. any amino acid is deemed a match to a wildcard).

Complexes are scored based on the total fragments that can be aligned to at least one allele in the complex, with a small penalty applied base on allele frequency in the population, a penalty for each allele included that was eliminated but subsequently recovered, and a bonus to boost scores of complexes with homozygous allele, and a penalty for solutions with wildcard characters which may cause spurious matches. The final score is given by:

```
Complex Score = AlignedFragments + FreqPenalty + HomBonus + RecoveryPenalty + Wildcard penalty

where

        FreqPenalty = 0.0015 * SUM[max(log10(Frequency),1e-4) * AlignedFragments]

   HomBonus = 0.0045 * (# of Homozygous alleles) * Fragments

   RecoveryPenalty = 0.005 * (# of Recovered alleles) * Fragments

    WildcardPenalty = 0.000015 * (# of wildcard characters in alleles) * Fragments
```

If 2 complexes are precisely equally scored, then the solution with the lowest alphabetical 4 digit allele types is chosen.

The matching unique, apportioned shared, and wildcard (in rare cases where the full allele is not present in the IMGT/HLA database) support for each allele is recorded in both tumor and normal.

As a further performance optimisation, if there are predicted to be more than 1 million complexes, then the evidence phase is first performed individually for complexes of 2 alleles per gene to find the top candidates for each of HLA-A, HLA-B & HLA-C. LILAC retains only the top 5 pairs including each individual allele candidate and then chooses the first 10 unique alleles appearing in the ranked list of pairs, with any common alleles also retained. The evidence phase is then subsequently run using this reduced set of candidate alleles.

Tumor and RNA status of alleles

Tumor allele specific copy number

LILAC optionally accepts a tumor .bam file and a gene copy number file (produced by PURPLE) which contains the minimum copy number and minimum minor allele copy number of each gene. If a tumor sample is provided, then fragments are counted for each allele in the determined type. For each of HLA-A, HLA-B & HLA-C, the minor allele copy number is assigned to the allele with the lowest ratio of supporting fragments for the allele in the tumor compared to the normal. The other allele for each is assigned the implied major allele copy number from the gene copy number file. If a gene is homozygous present in the normal sample, then the minor and major allele copy numbers are arbitrarily assigned in the tumor.

Somatic variant assignment to alleles

LILAC optionally accepts a .VCF input of somatic small indel and point mutations (called by Sage in the Hartwig pipeline) and can assign somatic variants to the specific allele which is damaged.

LILAC gathers the set of variants from the vcf (filter = “PASS”) that overlap either a coding or canonical splice region in any of HLA-A, HLA-B and HLA-C and finds all fragments that contain that variant. LILAC assigns portions of the fragment to the allele which matches the fragment at all heterozygous locations after excluding any somatic variants from the fragment. The allele with the highest matching fragment count is determined to contain the somatic variant. If the variant is assigned to 2 alleles with identical weight it is assigned with 0.5 weight to each. In the case of homozygous alleles, the variant is assigned arbitrarily to the 1st allele.

RNA expression of alleles

LILAC also optionally accepts a RNA bam. As per the fragments in the bam are counted for each allele in the determined type. This can be interpreted as a proxy for allele specific expression.

Benchmark

Germline-tumor agreement comparison

We assessed LILAC's robustness to perform *HLA-I* typing compared to two state-of-the art tools: Polysolver[2] (v4, *reference genome* hg19, *ethnicity* Unknown, *insertCalc* 0 and *includeFreq* 0) and xHLA[3] (with default parameters). We first retrieved GRCh37 aligned reads including the *MHC-I* locus (chr6:29,854,528-32,726,735) for the PCAWG and Hartwig germline and tumor samples. For each of these three tools, we first independently run the *HLA-I* typing for the germline and for the tumor across all available samples (see above, Hartwig and PCAWG cohort), and then we annotated whether there was a perfect agreement between them based on the inferred 2-field *HLA-I* haplotypes. Samples that failed to provide an output by any of the three methods were not included in the comparison. A total of 4,774 Hartwig and 2,099 successfully provided germline and tumor HLA haplotypes and were used in this analysis. Moreover, to reduce potential effects of tumor specific alterations on the *HLA-I* genes, we performed a similar comparison but limited to samples without *HLA-I* alterations (i.e. somatic mutations or LOH HLA events) according to LILAC.

Crosswise tools *HLA-I* haplotype comparison

We also assessed LILAC's agreement with two widely used tools for HLA typing: Polysolver (v4, *reference genome* hg19, *ethnicity* Unknown, *insertCalc* 0 and *includeFreq* 0) and xHLA (with default parameters). For each of these tools we first ran the germline HLA typing and we then performed the comparison based on the four digit *HLA-I* type annotation. Samples that failed to provide an output by any of the three methods were not included in the comparison. A total of 4,774 Hartwig and 2,099 successfully provided germline and tumor HLA haplotypes and were used in this analysis. We only considered that two, or three respectively, tools have an agreement if the four digit *HLA-I* alleles perfectly match among them.

HLA-I typing performance using Platinum and Yoruba family trios

The Illumina Platinum Genomes includes several family trios that underwent WGS and that had been extensively used by *HLA-I* typing tools to evaluate their sensitivity. Moreover, to include a family trio from a non-caucasian genetic ancestry we also processed the Yoruban family trio from 1000 Genomes. High-coverage raw sequencing data was downloaded from the original sources. The reference *HLA-I* types were extracted from the Additional File 1 of the Kourami manuscript[4].

HLA-I typing agreement with the TRACERx 100 lung cohort

After being granted access to the TRACERx lung WES cohort (EGAS00001002247) we downloaded the raw sequencing data of the germline and one representative tumor sample for the 100 patients included in the cohort. In total, 100 tumor samples were downloaded and subsequently underwent *HLA-I* typing by LILAC, resulting in 600 *HLA-I* typing calls.

As a reference for the comparison, we obtained, through personal communication with the authors; the *HLA-I* typing calls used in the LOHHLA original study[5]. Unfortunately, *HLA-I* typing calls for homozygous alleles were not available, which prevented their inclusion in the *HLA-I* typing analysis (i.e., that is the reason for matching 490 alleles in Fig. 1e instead of the 600). Moreover, the *HLA-I* typing originally provided by the authors missed a few samples. In those cases we processed an alternative tumor sample from the same patient with available *HLA-I* typing information. Finally, we considered a match by relying on 2-field allele resolution.

Copy number estimation of *HLA-I* compared to other genes

We aimed to evaluate whether the polymorphic nature of the *HLA-I* locus could have a negative impact on the tumor copy number estimation and subsequent annotation of *HLA-I* individual alleles. A proxy for incorrect tumor copy number estimation is the difficulty to assign an integer copy number. Therefore, we compared the proportion of samples with *HLA-I* genes with a purity adjusted integer copy number (i.e., estimated minor and major allele copy number <= 0.3 or >= 0.7) compared to other 1,000 randomly selected genes across the human exome. We performed this comparison across the two cohorts used in this study. Only samples with sufficient quality according to LILAC were used in this comparison.

LILAC's LOH of *HLA-I* agreement with LOHHLA in TRACERx cohort

To have an estimation on LILAC's ability to identify LOH of *HLA-I* we assessed the agreement with LOHHLA[5], a dedicated tool for LOH of *HLA-I* calling. LOHHLA was originally developed and tested using a high-coverage (>300x tumor sequencing depth) WES cohort[5] (TRACERx Lung). More recent studies have also applied LOHHLA to a set of colorectal WES/WES patients with high-sequencing coverage of their tumor samples[6]. However, according to our tests, the lower sequencing coverage of the PCAWG and the Hartwig cohorts did not seem to be suitable for LOHHLA, preventing its application in our dataset.

Instead, we decided to run LILAC on the TRACERx lung cohort (EGAS00001002247). Since identification of LOH of *HLA-I* by LILAC requires of the tumor copy number estimations (and the *HLA-I* typing already computed by LILAC, see above), we entirely re-processed 100 tumor-normal paired WES samples from TRACERx using our tumor analytical pipeline (https://github.com/hartwigmedical/hmftools). Several adjustments were made to adapt our pipeline, originally developed to work with WGS data, to the characteristics of this WES

dataset. Finally, the comparison was made by comparing the LOHHLA calls provided by the authors (personal communication) with LILAC's output.

Experimental validation by high-to allelic resolution HLA typing

We selected 96 Hartwig samples (10 from the tumor and 86 from the germline) to assess LILAC's agreement with an orthogonal approach based on high-to allelic resolution HLA typing (see below). The selection of samples were prioritized based on the following criteria: i) sample availability, ii) disagreement of LILAC with either xHLA or Polysolver, iii) challenging cases due to presence of rare alleles and iv) tumor samples bearing either somatic mutations or LOH of *HLA-I*. One germline sample failed to provide output with sufficient quality and was therefore not included in the final comparison (see Supp. Data 1).

HLA genes were amplified with the NGSgo® MX11-3 (GenDx) amplification strategy and libraries were prepared using the NGSgo® Library Full Kit (GenDx); both according to manufacturer's instructions. Libraries were sequenced on the MiSeq (Illumina) and the generated .fastq files were analyzed using the HLA typing analysis software NGSengine® (GenDx), 2.24.0, using the IMGT3.44 reference database. All data was reviewed by two independent reviewers and all exon heterozygous positions deviating from standard patterns were inspected and interpreted manually.

Application to Hartwig and PCAWG datasets

All pre-selected PCAWG and Hartwig samples (Extended Data Fig. 2a) were then processed by LILAC using the tumor-normal pair mode, which relied on the germline and tumor raw HLA-I files, the somatic mutation calls by SAGE (https://github.com/hartwigmedical/hmftools/tree/master/sage) and the copy number estimations by PURPLE (https://github.com/hartwigmedical/hmftools/blob/master/purple/) (both outputs were available as part of the Platinum Hartwig pipeline5 output).

All the pre-selected Hartwig samples but two (4,439 samples out of 4,441) were successfully processed by LILAC whereas 1,880 out of 2,275 PCWAG samples successfully achieved LILAC's quality control criteria. The main failure reason of PCAWG samples was insufficient coverage to perform a four digit HLA-I typing, and therefore they can not be further considered in this study.

As a result of the pipeline we obtained 2-field HLA-I class types for all successful samples alongside annotation of somatic mutations and the copy number estimations mapping for each allele.

Usage

To install, download the latest compiled jar file from the download links (https://github.com/hartwigmedical/tools/tree/master/lilac#version-history-and-download-links). Please check the github page for further information about how to use LILAC.

Supplementary Note 2: Neoantigen pipeline

Overview

The goal of this pipeline (named as Neo) is to provide a reliable collection of neoepitopes derived from tumor specific alterations. These alterations consider point mutations (i.e., missense variants and stop loss variants), small indels (i.e., in-frame indels and frameshift) and gene fusions (in-frame and out-of-frame fusions). The neoepitope pipeline works in 2 main steps to form a comprehensive set of neopeptide and neoepitope predictions from our DNA pipeline output:

- Determination of all novel peptides (i.e., neopeptides) from all point mutations, small indels and gene fusions.
- Calculation of allele specific presentation scores using a novel binding affinity prediction algorithm.

Although we annotate with expression information from RNA (when available), the neoepitope predictions are currently based solely on mutations found in the DNA. Hence we specifically ignore RNA events such as circular RNA, RNA editing, endogenous retroviruses and alternative splicing as we are unable to determine if these are tumor specific and hence will make neoepitopes. High confidence fusions detected in RNA but not found in DNA are also currently ignored. We also acknowledge that we miss protein level events including non-canonical reading frames, post translational amino acid modifications & proteasomal peptide splicing.

Workflow

1. Identification of candidate neoepitopes

We searched for potential neoepitopes for point mutations and structural variants that meet the following criteria (see below):

1. We included somatic point mutations and indels with coding effects (i.e., missense, frameshift, in-frame or stop loss) and a SAGE filter == ”PASS”.
2. We considered in-frame and out-of-frame gene fusions.

Type	Criteria
Point mutations	● Filter = ‘PASS’<br>● Coding effect in (missense, frameshift, in-frame indel and stop lost)

Fusions (intergenic or intragenic)	Rules as per LINX fusion calls with the following exceptions:<br>● The 5’ partner breakend for neo-epitopes MUST be in the coding region (exonic or intronic)<br>● No restriction applies on the coding context for the 3’ breakend for neoepitopes<br>● Fusions that are predicted to be terminated in the 5’ or 3’are not considered for neo-epitopes<br>● The 3’ transcript biotype for neo-epitope must not be ‘nonsense mediated decay’

Subject to the criteria above, all transcripts (or combination of transcripts in the case of fusions) are considered as candidate neoepitopes. Where 2 transcripts (or transcript combinations) lead to either the same amino acid sequence or the amino acid sequence of one transcript forms a subset of another, the transcripts are merged to form a single neoepitope. For each unique neoepitope, Neo outputs the amino acid (AA) sequence string broken up into ‘upstream’, ‘novel’ and ‘downstream’ segments as follows:

Field	Description
NeId	Unique Id for neoepitope
Variant type	One of: {MISSENSE, INFRAME, OUT_OF_FRAME_FUSION, INFRAME_FUSION, FRAMESHIFT}
VariantInfo	Unique identifier for variant<br>For point mutations = <chr>:<Position>:<ref>:<alt><br>For SV = <chrUp>:<posUp>:<orientUp>-<chrDown>:<posDown>:<orientDown>
GeneNameUp	Gene name for the upstream part of the neoepitope
GeneNameDown	Gene name for the downstream part of the neoepitope
UpstreamAA	Section of the neoepitope that matches the upstream transcript
DownstreamAA	Section of the neoepitope that matches the downstream transcript (if any)
NovelAA	Novel section of the neoepitope (if any)
WildtypeAA	Wildtype AA sequence for agretopicity calculation (missense variants only)
UpTranscripts	List of transcripts in the up gene that support the neoepitope
DownTranscripts	All unique transcripts on the up gene that support the neoepitope

Note that the precise definition of the novel segment and upstream and downstream AA depend on the type of event. The exact rules are outlined in the table below:

Variant Type	Novel Segment	Upstream flank	Downstream flank
Missense SNV/MNV*	Ref->Alt AA(s)	Up to 16 AA limited by start codon	Up to 16 AA limited by stop codon
Inframe*	If conservative inframe, inserted AA only else also use flanking disrupted AA on each end	Up to 16 AA limited by start codon	Up to 16 AA limited by stop codon
Stop_lost / frameshift*	All downstream AA until	Up to 15 AA limited by	NA

	new stop codon reached	start codon	
Inframe fusion (Phase=0)	NA***	Up to 16 AA limited by start codon	Up to 16 AA limited by stop codon
Inframe fusion (Phase = {1,2})	Mixed transcript AA***	Up to 16 AA limited by start codon	Up to 16 AA limited by stop codon
Out of frame coding to coding or coding to<br><br>non-coding fusion	Possible mixed transcript AA + all downstream AA until new stop codon eached**	Up to 16 AA limited by start codon	NA**

* Where multiple somatic variants are phased within 17 AA, include entire intermediate section as novel AA
**For coding to 5'UTR fusions, if a start codon is reached prior to a novel stop codon and is 'inframe', the novel segment should be limited to the region up to the new stop codon with the downstream flank set as the first 17 AA of the 3' partner.
*** For exonic-exonic fusions, include any inserted sequence

Neo further annotates each of the candidate neoepitopes with TPM and direct RNA fragment support for the novel amino acid sequence. TPM per transcript is sourced from Isofox (https://github.com/hartwigmedical/hmftools/tree/master/isofox) if RNA-Seq is available. If not available, it is estimated as the median of the cancer type or full cohort where cancer type is not known. Neo also reanalyses the RNA BAM to count the RNA depth at the location of the variant that caused the neoepitope and the direct RNA fragment support for the neoepitope (defined as matching precisely the 1st novel AA and 5 bases either side).

2. Calculation of allele specific binding affinity and presentation scores

Using the identified neoepitopes, we determine all candidate peptide and allele pairs (pHLA) combinations that may be presented by the cell. For each candidate neoepitope, we consider all peptides between 8 and 12 length which either overlap the novel amino acid sequence or overlap both the upstream and downstream amino acid sequence.

For each pHLA we estimated a presentation likelihood based on a newly developed Position Weighted Matrix (PWM) algorithm that considers both the binding affinity and the processing likelihood for each pHLA pair.

2.1 pHLA algorithm description

2.1.1. Conventions regarding positions, peptide lengths and HLA allele binding motifs

Position Independence

Our model assumes that the peptides at each position each have an independent impact on binding and that no correlated effects apply (an assumption held almost universally across binding prediction tools). In fact, a preliminary internal test showed that this assumption holds true.

Peptide length mappings

Neo supports 8-12 length kmers. Our model assumes that anchor (2nd and last peptide position) positions and their surrounding positions have high similarity across peptide length with central peptides variable and relatively less important for binding. We therefore convert all peptides to a 12mer, with the following padding conventions for shorter peptides.

**12mer**	0	1	2	3	4	5	6	7	8	9	10	11
**11mer**	0	1	2	3	4	X	5	6	7	8	9	10
**10mer**	0	1	2	3	4	X	X	5	6	7	8	9
**9mer**	0	1	2	3	4	X	X	X	5	6	7	8
**8mer**	0	1	2	3	4	X	X	X	X	5	6	7

Flanking sequences

The flanking amino acids upstream and downstream are known to impact cleavage and proteasomal processing. To capture these impacts we include 3 upstream amino acids (U3,U2,U1) and 3 downstream amino acids (D1,D2,D3) in the model. The enrichment/depletion of amino acids at these positions globally (including 'X' where the flanking sequences are beyond the start or end of an allele) is included in the peptide score.

Binding Motifs

We utilize the same assumptions as NetMHCpan[7] which is that only proximate (within 4 angstroms across a representative set of HLA-A/ HLA-B structures) polymorphic residues may affect binding, which yields 34 distinct positions with the following specificities.

Position	Proximate Polymorphic Amino Acid Residues
0	31,83,86,87,90,183,187,191,195

1	31,33,48,69,86,87,90,91,94,123,183
2	94,121,123,138,180,183
3	90,182,183,187
4	93,94,182
5	93,94,97,98,121,180
6	93,97,121,138,171,174,176,180
7	97,100,101,171
8	98,101,104,105,108,119,121,140,142,167,171

In general we observe that positions with identical binding motifs observe highly similar amino acid weight distributions in mass spectrometry observations.

Binding Motif similarity

We assume that binding motifs with similarity tend to have similar bindings. We estimate binding motif similarity by summing the log likelihoods from the BLOSUM62 substitution matrix across all binding positions.

2.1.2. Training dataset

We have curated IEDB[8] and the literature for high quality unbiased monoallelic mass spectrometry results assessing that **i)** the datasets were monoallelic and **ii)** whether the study did not appear to contain an empirically high rate of likely false positive results. Binding affinity results are not used in the training data due to their inherent experimental selection bias. Overall we identified 20 studies (all included in IEDB) with 413,000 MS observations across 103 alleles to be used in our training set. For 8 specific alleles which had no mono-allelic results, but where there was sufficient high quality multi-allelic data (specifically A*69:01,B*35:08,B*41:01,A*26:08,C*15:05',B*44:09,B*44:27 and B*44:28) we included the results from 4 additional studies.

Finally, we also included monoallelic data from the recent Pyke et al.[9] (hereafter also referred to as Sherpa dataset) study which has results from 25 monoallelic cell lines including 6 additional cell lines not represented in the IEDB data. To eliminate potential experimental artifacts, the Sherpa dataset was also filtered to include only peptides that were found to be bound to 2 or less distinct alleles.

Any {peptide;allele} observations found in more than 1 dataset may be counted multiple times towards position matrices. We also have determined the 3 bases flanking both upstream and downstream for each peptide where possible by matching the peptides to th

reference proteome. 10% of the full data is held back as a validation dataset (see Validation section).

2.1.3 Construction of Position Weight Matrix (PWM)

A position weight matrix is constructed per allele per peptide length. To deal with sparsity of data for specific alleles and peptide lengths, we use the following principles to learn from other peptide lengths and alleles:

1. Peptide lengths may learn from other lengths when they lack sufficient allele and length specific data. The more similar the peptide length, the higher the weighting

2. Alleles may learn from other alleles with identical or similar binding motifs for that position when they lack sufficient allele specific data. The more similar the binding motif, the higher the weighting

This is implemented in a 2 step process. First we consolidate all specific length counts into a single length weighted count (LWCount) for the tested peptide length for each allele using the following formula:

```
LWCount(A,L,P,AA) = Count(A,L,P,AA) + LWF * SUM(l<>L)
[Count(A,l,P,AA) /abs(L-l)] * maxLW / max( LWF *
SUM(l<>L)[Count(A,I,P) /abs(L-l)],maxLW)
```

Then, using the motif m of the tested allele, we consolidate all matching and similar binding motif observations from other alleles to obtain a total weighted count (WCount) using the following formula:

```
WCount(A,L,P,AA) = LWCount(A,L,P,AA) + MWF* SUM(a<>A) [
LWCount(a,L,P,AA) * (2^(LogSim(m,M)) / MAX(i=all
motifs)[2^(LogSim(i,M))]] * maxMW / max(MWF * SUM(a<>A) [
LWCount(a,L,P) * (2^(LogSim(m,M)) / MAX(i=all
motifs)[2^(LogSim(i,M))]],maxMW)
```

where:

- A=allele
- L= length
- P= Peptide position
- AA = amino acid
- M = binding motif of allele A at position P
- LWF = length weight factor (<=1; default = 0.25)
- MWF = motif weight factor (<=1; default = 0.25)
- maxLW = max length weight (default = 200)
- maxMW = motif weight factor (default = 200)
- Obs(a,l) = observations of peptides of binding allele a and peptide length l

- LogSim(a,b) = Blosum62 log similarity of motifs a and b summed over all motif positions

The output of this is a final weighted position weight matrix for each allele and length.

2.1.4. Scoring and ranking per allele per peptide length

Each peptide is scored based on the positional amino acid frequencies relative to the amino acid frequency in the proteome:

```
PeptideScore = Sum[Log2(max(P(x,i),0.005)/Q(x))]
```

where

- P(x,i) = % weight of amino acid = x at position = i (note for C we use 3*weight to correct for MS bias)
- Q(x) = frequency of amino acid = x in the proteome

An additional flank score based on a pan allele flanking PWM is calculated as follows:

```
FlankScore = Sum(i=U3-U1,D1-D3)[Log2(P(x,i)/Q(x))]
```

As has been noted previously by other groups, we do observe enrichment when U1 = ‘M*’ (ie the first amino acid of the coding sequence) or D1 = ‘X’ the stop codon of a transcript, but not for the other bases in the flanks. Hence for the U1 and D1 base we set the PWM score to be the observed enrichment for ‘M*’ (approximately 2 fold greater) and ‘X’ (approximately 4 fold greater) respectively. For the other 2 flanking bases on either side we observe no further enrichment in ‘M*’ or ‘X’ and hence set the PWM score simply to 0 (no enrichment or depletion) if they overlap the start or end of the transcript.

The total score is then set to:

`TotalScore = PeptideScore + FlankScore`

The score is converted to a binding rank percentile (per allele per peptide length) by comparing to the percentile scores compared to scores for 100,000 peptides of the same length randomly from the proteome (note that random peptides are excluded if they are found to be binders already in the MS results). Where flanks are available the rank is given relative to the total score distribution and where not available relative to just the peptide score distribution.

2.1.5. Relative presentation likelihood and ranking per allele (pan peptide length)

To assess the relative likelihood of presenting peptides of different lengths for a particular allele given the binding ranks, we determine the relative density of mass spectrometry observations in our training set per peptide length per binding rank percentile bucket. The relative likelihood is calculated as:

```
RelPresentationLikelihood =
MSobs(A,Rb,L)/Size(Rb)/SUM(Rb,L)[MSobs(A,Rb,L)/ Size(Rb)]
```

Where

- A = Allele
- L = Peptide Length
- Rb = Rank Bucket. Exponential buckets in powers of 2 are used to reflect the relative importance of the very low binding ranks: {0.00005,0.0001,0.0002,0.0004,...,0.8192}

To deal with sparse MS data, particularly for non-9mers, the density of a given ranking bucket is set to be at least as high as any lower ranked bucket. Furthermore, so that we can always rank the higher buckets regardless of the number of observations, any bucket with 0 observations is padded with observations of min(totalObservationsPerAllele/1000,0.25) for bucketed rank < 1%, or half the observations of the preceding ranked bucket where the bucketed rank > 1%.

The output of this algorithm is a set of weights per bucket per peptide length reflecting the relative likelihood of an observation from that bucket being presented on the surface in that cell. For individual peptides we can predict an exact relative likelihood by interpolating the rank between the bucketed values.

An example of the output for A*29:02 is shown below indicating that a 9mer with 0.00005 rank is ~28x (ie 0.3007/0.0116) more likely to be presented than an 8mer with the same rank and ~6x (ie 0.3007/0.0520) more likely to be presented as a 9mer with a rank of 0.0004.

	Bucketed Rank	MS Obs by allele by length by ranking					Relative Presentation likelihood				
		8	9	10	11	12	8	9	10	11	12
A2902	0.00005	14	364	100	89	36	0.0116	0.3007	0.0826	0.0735	0.0297
	0.0001	8	402	67	61	26	0.0033	0.1661	0.0277	0.0252	0.0107
	0.0002	1	438	52	44	6	0.0009	0.0905	0.0125	0.0091	0.0014
	0.0004	9	504	121	50	14	0.0009	0.0520	0.0125	0.0052	0.0014
	0.0008	10	656	118	35	18	0.0006	0.0339	0.0061	0.0018	0.0009
	0.0016	23	691	146	41	19	0.0006	0.0178	0.0038	0.0011	0.0005
	0.0032	28	492	166	44	19	0.0004	0.0064	0.0021	0.0006	0.0002
	0.0064	28	382	99	38	7	0.0002	0.0025	0.0006	0.0002	0.0000
	0.0128	17	323	95	22	12	0.0001	0.0010	0.0003	0.0001	0.0000
	0.0256	20	166	55	11	1	0.0000	0.0003	0.0001	0.0000	0.0000
	0.0512	12	93	19	5	3	0.0000	0.0001	0.0000	0.0000	0.0000

The relative presentation likelihood is calculated for each 4-digit and 2-digit allele with more than 200 MS observations in the training data as well globally for HLA-A, HLA-B and HLA-C. Any 4-digit allele which does not have sufficient MS observations is assigned the likelihoods of the 2-digit allele if available or if not the likelihoods for the HLA gene as a whole.

To conclude, a presentation percentile rank is calculated for each allele across all lengths by comparing the relative likelihood of the peptide compared to that of all negative decoy

peptides (equally weighted across 8,9,10 and 11-mers to give best compatibility to other tool rankings).

Each pHLA score was then ranked compared to a random set of 100,000 peptides derived from the human canonical proteome to derive a presentation likelihood rank for each pHLA.

3. Expression adjusted presentation likelihood algorithm

Multiple studies have shown the importance of including RNA expression in the prioritization of neoepitopes. The pHLA presentation scores were further adjusted by the inclusion of the normalized RNA expression of the mutated transcript/s (including the 5’ and 3’ transcripts in gene fusions). When RNA expression was not available, we used the average expression across the patient cancer type or the pan-cancer when the cancer type is unknown. We then calculated the expression-adjusted likelihood rank (ExpLikelihoodRank) for each pHLA using the expression adjusted likelihood compared to the same randomly selected peptides.

3.1. Training data

For training the impact of expression on presentation likelihood we use the subset of the mass spectrometry training dataset included with the HLAthena publication[10] together with the matched TPM estimates provided with this publication for the B.721.221 cell line. For the purpose of the training the TPM of the gene is assumed to be the TPM of the transcript containing the epitope.

3.2. TPM adjusted likelihood rank

To determine the impact TPM expression has on presentation we compared the TPM of the MS identified peptides of strong predicted binders (LRank<0.1%) found to be presented in the HLAthena training data to all predicted strong binders from the proteome for the same alleles. For each $\log_2$ TPM bucket we calculate the proportion of pHLA combinations that are found to be presented. We find this to be a very strong relationship, ranging from a <<1% chance of presentation where TPM < 1 up to higher than 20% chance where TPM > 1000:

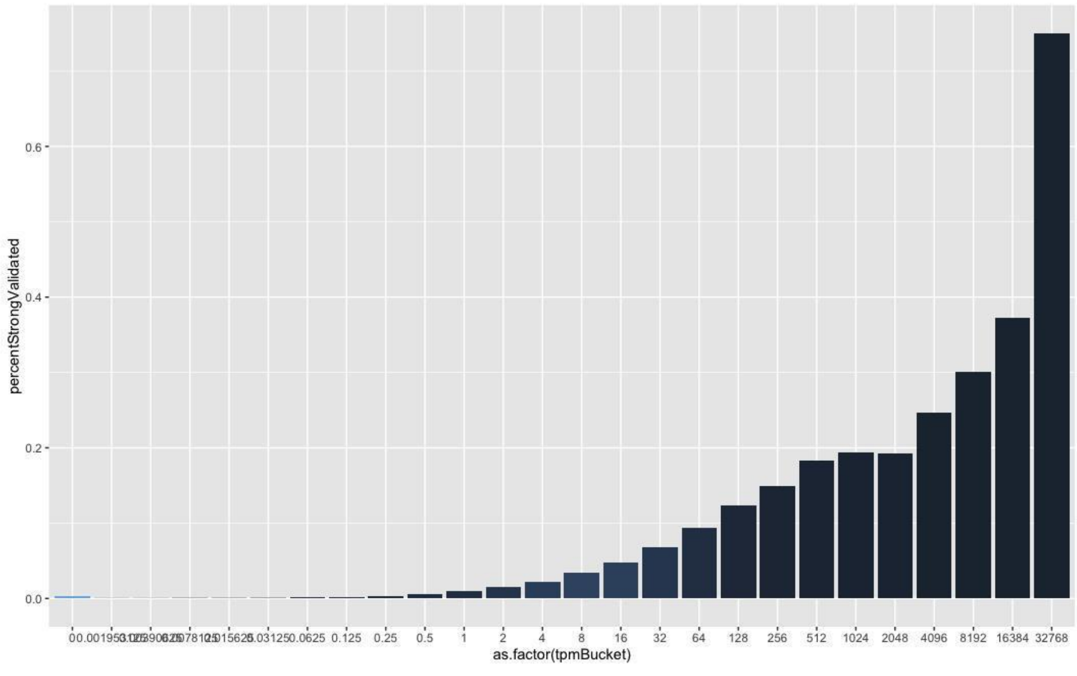

Using this observed rate of TPM we can calculate:

```
ExpAdjLikelihood = PresentationLikelihood * TPMLikelihood /
[PresentationLikelihood * TPMLikelihood +
(1-PresentationLikelihood) * (1-TPMLikelihood)]
```

We then calculate a new overall rank globally across all pHLA using the expression adjusted likelihood compared to the same randomly selected peptides.

Neoepitope identification in Hartwig and PCAWG dataset

We defined the collection of neoepitopes as those pHLA with a LikelihoodRank < 0.02 and ExpLikelihoodRank < 0.02 (i.e., within the 2% percentile of all peptides). Hence, the total neoepitope load of a patient tumor sample is the sum of all pHLA neoepitopes (using the germline's HLA-I types) with LikelihoodRank < 0.02 and ExpLikelihoodRank < 0.02.

Neoepitope clonality

Each predicted neoepitope (see above) derived from point mutations and small indels was matched with the source variant estimated clonality from PURPLE. We defined clonal mutations as those with a subclonal score lower than 0.85. Gene fusions were not considered for this analysis.

Neo pipeline validation

We assessed the performance of our neoepitope prioritization pipeline (Neo) by conducting four orthogonal validations:

1. Neo performance compared to MHCFlurry2.0 in a curated dataset of experimentally identified peptides derived from HLA monoallelic cell lines.
2. Neo ranking of random peptides.
3. Neo robustness by performing an allele leave-one-out experiment.
4. Neo expression adjusted model performance compared to the non-expression model.

For these validations, we assumed that the 100,000 random peptides used for the ranking likelihood calculation (after filtering any {peptide,allele} combinations included in the training data) were not binders. This is a conservative assumption as some of the highest ranked random peptides would certainly be expected to bind. Therefore, since only the top ~0.1% of peptides are expected to strongly bind, we mostly used the True Positive Rate (TPR) as the performance metric. This metric measures how well the model is able to rank known presented peptides (from a curated set) compared to the random set of 100,000 peptides. Other measurements, such as AUC measurements may be dominated by the relative performance of very weak predictions and are therefore not as informative in our endeavor.

1. Neo performance compared to MHCFlurry2.0

The aim of this validation is to compare our pipeline ability to prioritize bona-fide peptides presented by the HLA-I in comparison to an outstanding open access tool such as MHCFlurry2.0[11]. To do so, we held-out 10% of the training set composed by the non-redundant union of experimentally identified peptides reported in IEDB and in other studies (see above Training set section). Hence, this 10% was not used in our training and will be used as a validation dataset to evaluate the True Positive Rate (TPR) across multiple rank thresholds compared to MHCFlurry2.0. The TPR was estimated as the number of predicted peptides below the given threshold compared to the total number of peptides in the validation dataset. Moreover, to ensure comparability we use the presentation percentile of MHCFlurry2.0 and then recalculated a rank using 100k random peptides in the same manner we did for the Neo Likelihood ranks. This number deviates from the MHCFlurry Presentation rank which may have different assumptions about negatives

The average TPR was higher in Neo predictions compared to the predictions from MHCFlurry2.0 by relying in six ranking thresholds ($1e^{-06}$, $1e^{-05}$, $1e^{-04}$, $5e^{-04}$, $1e^{-03}$, $1e^{-02}$, $2e^{-02}$ and $1e^{-01}$, see Figure1 below). This trend was maintained when we split by peptide length from 8-kmers to 11-kmers (Figure 1). Notheworthly, the greatest differences in TPR were observed for 8-mers (average TPR Neo 0.48 compared to 0.32 of MHCFlurry2.0), while the difference for other peptide lengths was considerably lower. Taken together our results showed that Neo sensitivity to rank true binders is slightly better than MHCFlurry2.0 at different percentile rank thresholds.

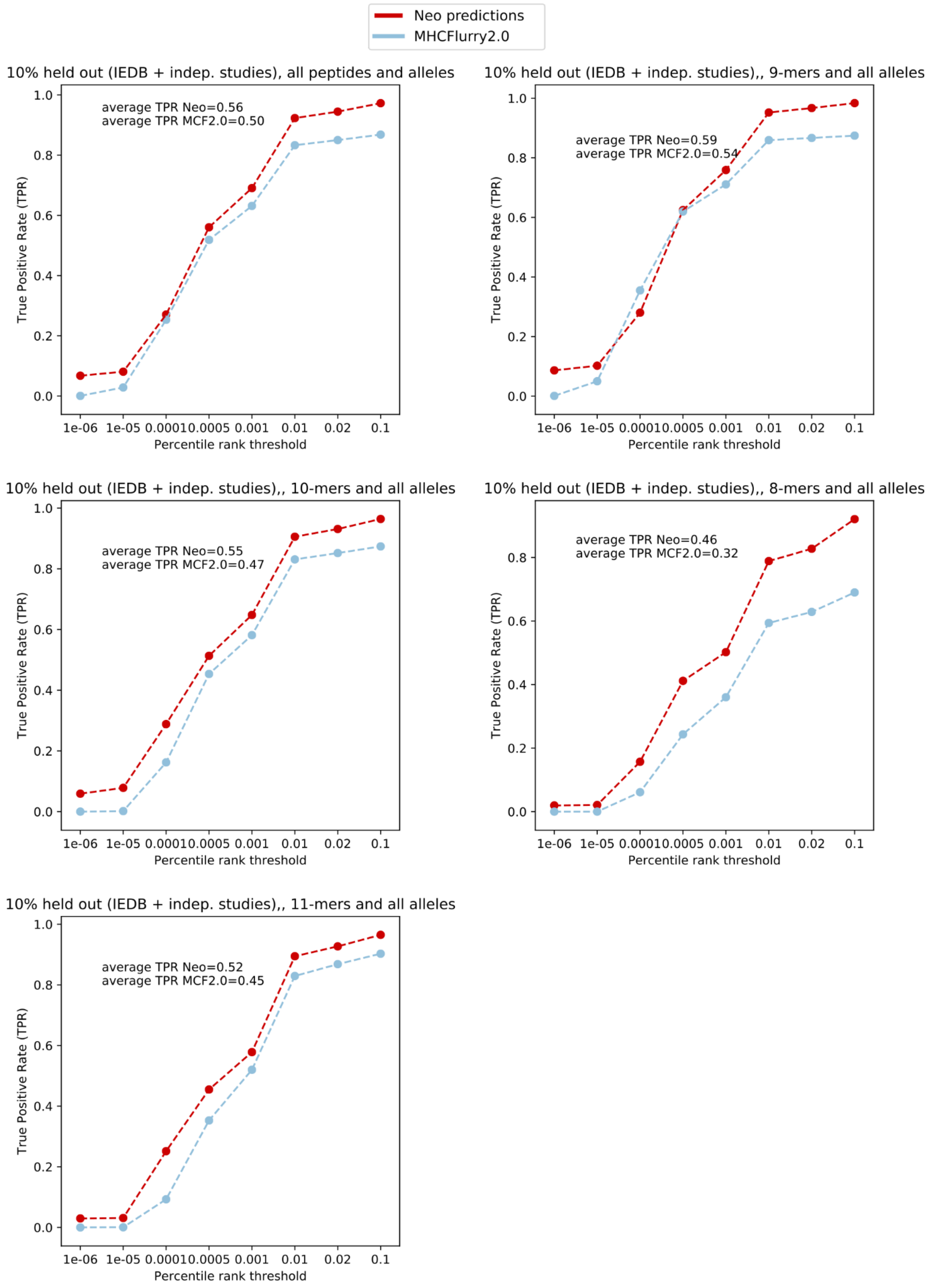

Figure 1. Neo performance in a 10% held-out validation dataset compared to MHCFlurry2.0. The x-axis represents the threshold of the ranking used as predicted peptides. The y-axis represents the TPR at each threshold. Red and blue dots and lines represent Neo and MHCFlurry2.0 performance, respectively.

2. Neo ranking of random peptides.

In the previous analysis we have shown Neo's sensitivity to rank true binders among the lowest percentile ranks. However, whether this is a unique feature of true binders or a systemic bias towards low percentile rankings is yet unclear. To address this, we evaluated Neo median percentile ranking in a set of 50k random peptides at five peptide lengths (from 8-12mers, 10k peptides per kmer length).

Reassuringly, we observed that the median allele percentile ranking distribution was very close to 0.5 (ie., percentile rank 50th) across all evaluated lengths and alleles (Figure 2). These results show that observed percentile rankings for true binders are not the effect of a systemic bias towards low rankings and are thus the result of Neo's ability to discern between true binders are random peptides.

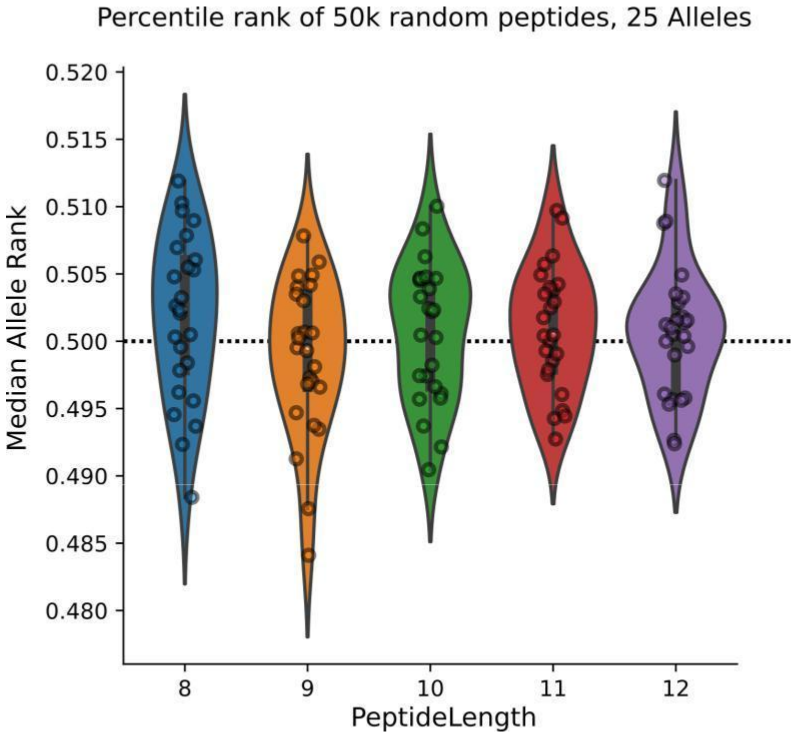

Figure 2. Neo median percentile ranking in a set of 50,000 random peptides across 25 HLA-I alleles. Each dot represents the median percentile rank across the evaluated random peptides for a particular allele and peptide length. Dashed horizontal line represents the 50th percentile ranking.

3. Neo robustness by performing an allele leave-one-out experiment.

We aimed to assess how well our model is able to rank the presentation of peptides for an HLA allele that lacks representation in the training set. To do so we performed the following steps:

I. For each of the 116 HLA alleles with sufficient representation in the training dataset we iteratively removed the peptides from the curated dataset and re-trained the full model (i.e., leave-one-out experiment).
II. We computed the TPR at a 0.02 ranking threshold on the curated peptides for that allele (i.e., number of ranked peptides with a ranking likelihood below that threshold compared to the total number of peptides for that allele in the original training set).
III. We compared the leave-one-out TPR to the original TPR by training with the full dataset. The percentage of TPR decrease (%TPR loss) measures the ability of our model to generalize predictions for HLA alleles that lack representation. Alleles with high %TPR loss are those for which the model can not reliably make predictions without the training data. Conversely, HLA alleles with low %TPR loss are those for which the model can find accurate predictions by generalizing the predictions from other alleles with available training data.

We observed an average %TPR loss of 4.4% ± 6% std. (Figure 3). Only 15 HLA alleles (~12% of the 116 screened alleles) show a %TPR loss greater than 10% (Figure 3), highlighting the capacity of Neo pipeline to generalize predictions for alleles lacking representation in the training set. Certain alleles such as B*08:01, B*15:03 or A*30:01 displayed a very high %TPR loss suggesting that they may have a unique binding preference that can not be interpreted from neighbor HLA alleles (Figure 4). Another plausible explanation is that the 34 HLA amino acids selected for our model are unable to capture the binding preference of these alleles and additional amino acids are thus needed.

When splitting by peptide length, we observed that the most consistent predictions were for 9-mers, likely due to the higher representativeness of these peptides in the training dataset. Other k-mers had greater average %TPR loss.

Taken together our results show that, in general, our pipeline is able to accurately rank peptides for HLA alleles lacking representation in the training set and that our learn-from-others approach is a robust strategy to prioritize peptides presented by the HLA complex.

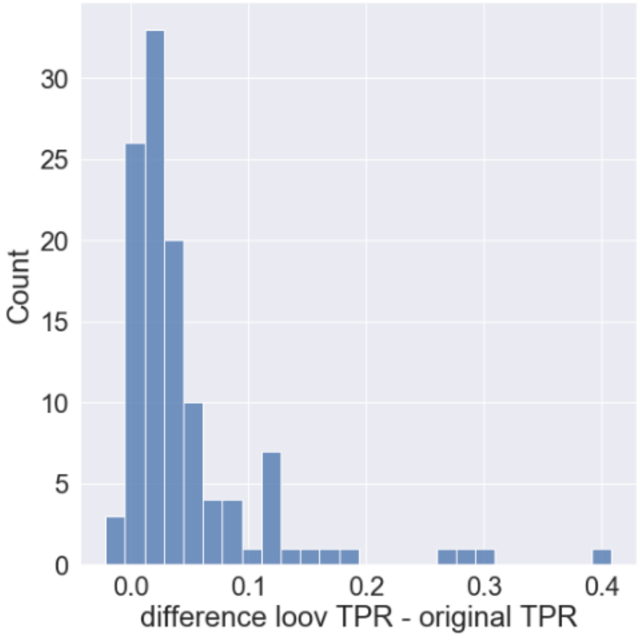

Figure 3. Percentual TPR loss distribution after leave-one-out

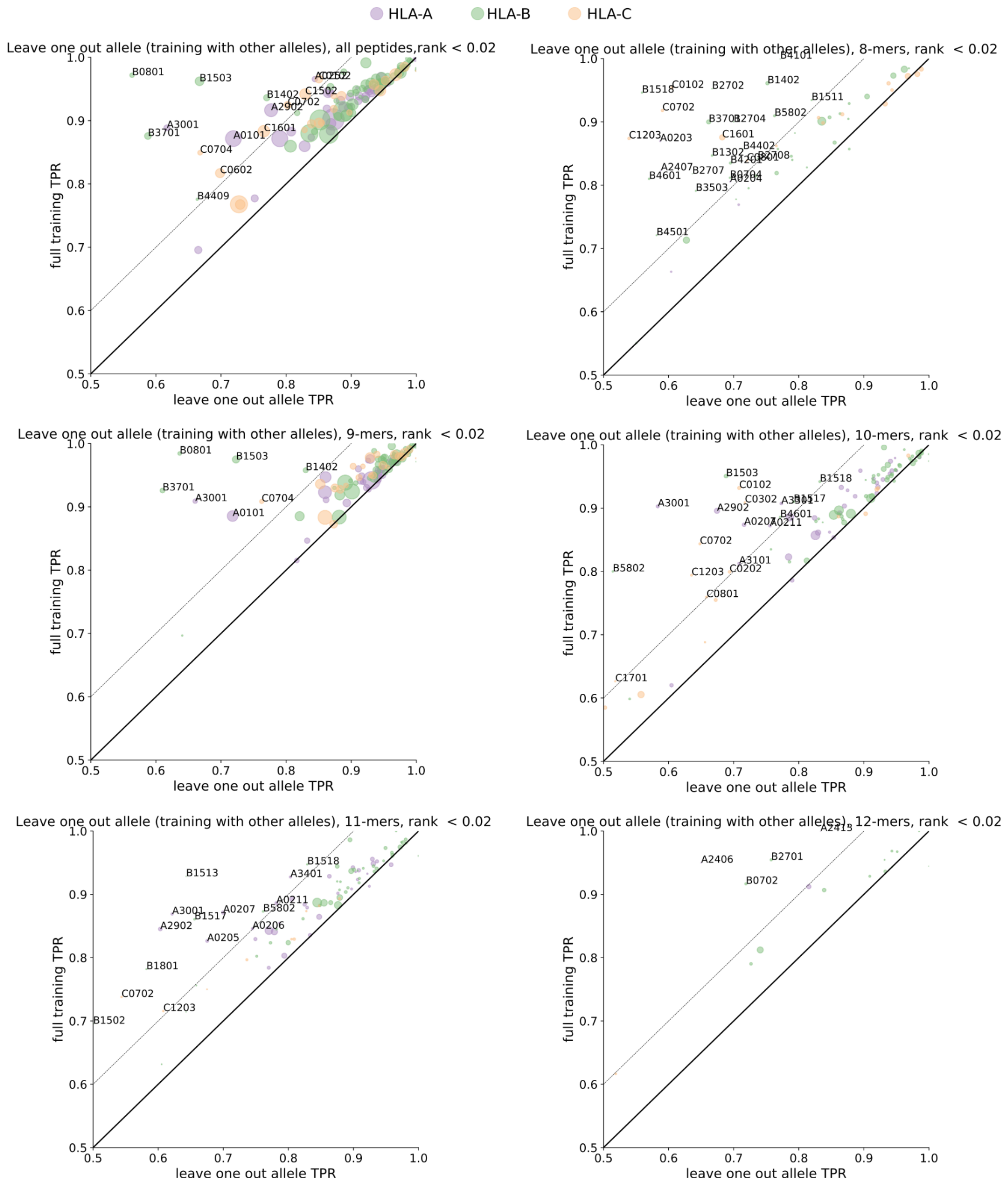

Figure 4. HLA allele leave-one-out analysis. The X-axis represents the leave-one-out allele TPR (by removing the allele peptides of the training set and re-training the model). The Y-axis represents the original TPR including all peptides in the training set. Thick continuous line overlaps with the diagonal. Dashed lines represent a %TPR loss greater or equal to 10%. HLA alleles with the annotated label are those with a %TPR loss greater than 10%.

4. Neo expression adjusted model performance

We evaluated whether the expression adjusted model (see above Expression adjusted presentation likelihood) is able to improve the predictions from the expression naive model. To assess this, we leveraged the part of our training set that was obtained from ref.[10]. Briefly, this is a publicly available dataset that contains experimentally identified peptides through immunopeptidomics across 95 HLA monoallelic cell lines engineered from B721.221. We therefore matched these observations with the RNA-seq expression of the source HLA-null cell line B721.221.

We observed that the model adjusted by the RNA expression has consistently higher TPR across all the evaluated ranking thresholds ($1e^{-06}$, $1e^{-05}$, $1e^{-04}$, $5e^{-04}$, $1e^{-03}$, $1e^{-02}$, $2e^{-02}$ and $1e^{-01}$, see Figure5 below). The average TPR across these thresholds was higher in the expression adjusted group (0.52) compared to the non-adjusted (0.45). Taken together, these observations confirm the added value of adjusting by the RNA expression of the source transcript. Unless otherwise specified, this model will be used to select the potential (neo)epitopes derived from tumor specific alterations in our neoepitope prioritization pipeline.

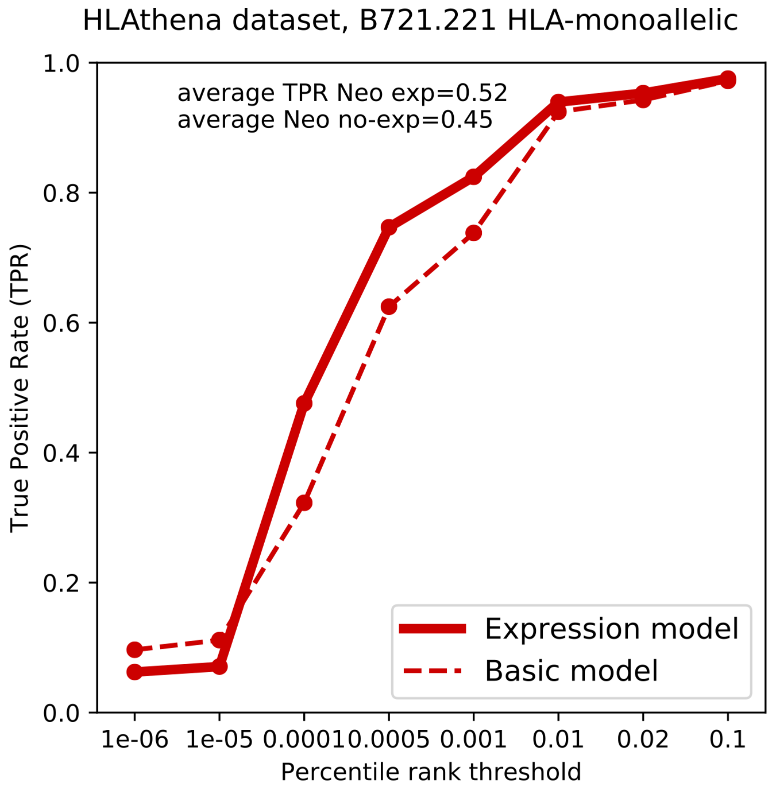

Figure 5. Performance comparison of expression adjusted versus non adjusted models. The x-axis represents the evaluated percentile ranking thresholds. The y-axis represents the TPR at the given thresholds. Basic model is the non-expression adjusted model.

Usage

Neo use the following inputs from the Hartwig pipeline

- **Somatic variants:** PURPLE somatic .vcf
- **Structural variants:** LINX candidate fusion neoepitopes (a new file produced by LINX to output all candidate neoepitope files).
- **HLA typing:** LILAC output (or from alternative methods as long as they are formatted appropriately).

Where RNA is available additional annotations are added, an effective TPM of each neoepitope is also estimated based on the following inputs

- **Gene Expression:** Isofox (https://github.com/hartwigmedical/hmftools/tree/master/isofox) transcript expression
- **Neo-epitope fragment support:** RNA .bam

For presentation and immunogenicity predictions, Neo also uses a number of resource files, that are pre-calculated from external resources (including IEDB[8], the HLAthena[10] publication and the IPD-IMGT/HLA[1] database). See https://github.com/hartwigmedical/hmftools/tree/master/neo for more information about how to use Neo.

Supplementary Note 3: GIE and tumor genomic features

Tumor genomic features and GIE association

We aimed at identifying cancer type genomics features associated with an increased GIE risk. Hence, we performed a cancer type aggregation of the two datasets (i.e., metastatic and primary) to increase statistical power of the analysis. We then computed 99 genomic features and 366 driver alterations and evaluated its association with GIE across 38 cancer types with sufficient representativeness (i.e., total number of samples >=15). For this analysis, we used a definition of GIE that considers all GIE events except non-focal LOH of *HLA-I* as it likely reflects a passenger event in tumor evolution (see Fig. 6 of main text).

Background simulation of GIE alterations

Some of the identified associations between tumor genomic features and GIE incidence could be explained by higher background mutation and CNV rates. In order to distinguish between those tumor genomic features exclusively associated with GIE and those that are likely the result of higher background alteration rate we devised a background control by performing 100 simulations of GIE alterations (i.e., GIE simulations) across the tumor samples included in this study. More in detail, we followed the next steps:

1. We randomly selected 100 genes from the entire human genome (excluding driver cancer genes, genes in sexual chromosomes and GIE genes).
2. Then we performed 100 GIE simulations following the next steps:
    a. For every simulation, s_i ($i \in 1..100$), we randomly sampled with replacement 21 genes from step 1) (i.e., equal to the total number of genes included in the study).
    b. Next, these background 21 genes were matched with any of the GIE pathways included in the study by preserving the number and proportion of genes originally associated with every pathway (i.e., three for *HLA-I*, eight for antigen presentation, seven for IFN-γ, one for CD58, etc.). In that manner, every GIE gene from the study has a randomly selected decoy gene in the simulation s_i.
    c. Then, for every decoy gene (labeled with a GIE pathway in b.) we annotated the presence of alterations considered in that specific pathway across all samples included in the study. For instance, if the decoy gene was linked to the antigen presentation pathway, we considered as mutated those samples with monoallelic truncating variants, biallelic non-synonymous mutations or deep deletions. The only exception was the LOH and deep deletions of *HLA-I* genes, for which, given the genomic proximity of the three genes (*HLA-A*, *HLA-B* and *HLA-C*), we only considered one decoy gene that would act as a proxy of LOH (or deep deletion, respectively) in the *HLA-I* locus.
3. Then for every tumor sample and every simulation s_i ($i \in 1..100$) we annotated the existence of simulated GIE alterations if there was any GIE alteration across the six simulated GIE pathways in that specific sample.

In the association between tumor genomic features and GIE we only considered as exclusively associated with GIE those genomic features that showed a significant association (i.e., q-value < 0.05) that did not show a simulated GIE association (i.e., less or equal than 2% of GIE simulations showed a significant association with the same genomic feature). To perfectly match the definition of GIE used in the genomic features GIE association analysis (see above), we also excluded non-focal LOH of *HLA-I* (i.e., its decoy gene in each simulation) as part of the GIE simulations.

Tumor mutation burden, neoepitope load and SV load

For each cancer type, we used a univariate logistic regression to quantify the association of 21 TMB-related measurements (see Supp. Data 4 for full list of evaluated features) and the presence/absence of a GIE event. Independent variables were z-scored. The *Logit()* function (with default parameters) from the statsmodels[12] v.0.13.1 library was used to perform the logistic regression. This function provides the odds ratio with confidence intervals alongside the p-value of significance. The p-values were adjusted with a multiple-testing correction using the Benjamini–Hochberg procedure (alpha=0.05).

The clonality of each variant was defined using the PURPLE subclonal likelihood estimation. More specifically, a variant was considered as clonal if the estimated subclonal likelihood was lower than 0.85.

The global neoepitope load of each patient's sample was calculated as the sum of the predicted neoepitopes (i.e., allele specific neoepitope repertoire, see Supp. Note 2) across the germline *HLA-A* alleles inferred by LILAC. The subset of neoepitopes (i.e., fusion derived, mutation derived, clonal and subclonal) was computed by matching the source alteration -and their clonality- of each predicted neoepitope. Therefore, a mutation (or gene fusion) may be the source for multiple neoepitopes.

Mutational signatures

The number of somatic mutations falling into the 96 single nucleotide substitution (SBS), 78 double base substitutions (DBS) and 83 indel (ID) contexts (as described in the COSMIC catalog[13] https://cancer.sanger.ac.uk/signatures/) was determined using the R package mutSigExtractor (https://github.com/UMCUGenetics/mutSigExtractor, v1.23).

SigProfilerExtractor (v1.1.1) was then used (with default settings) to extract up to 21, 8 and 10 de novo mutational signatures for SBS, DBS and indels (respectively). This was performed separately for each of the 22 tissue types which had at least 30 patients in the entire dataset (aggregating primary and metastatic samples, see Supp. Table 2). Tissue types with less than 30 patients as well as metastatic patients with unknown primary location type were combined into an additional 'Other' group, resulting in a total of 23 tissue types for signature extraction. In order to select the optimum rank (i.e. the eventual number of signatures) for each tissue type and mutation type, we manually inspected the average stability and mean sample cosine similarity plots output by SigProfilerExtractor. As a result, there were 484 *de novo* signature

profiles extracted across the 23 tissue type groups (see Supp. Table 2 and Supp. Data 4). Least squares fitting was then performed (using the fitToSignatures() function from mutSigExtractor) to determine the per-sample contributions to each tissue type specific *de novo* signature.
The extracted *de novo* mutational signatures with high cosine similarity (>=0.85) to any reference COSMIC mutational signatures with known cancer type associations[13] were labeled accordingly (number of labeled *de novo* signatures = 274 matched to 57 COSMIC references).

For the collection of remaining non-labeled *de novo* signature profiles of each mutation type, we reasoned that there could be one or more signatures that are highly similar to those found in the set of signatures of other tissue types (and thus likely representing the same underlying mutational process) and that have not been yet matched to a COSMIC reference. We therefore performed clustering to group likely equivalent signatures and to label them as such. Specifically, we followed the next steps:

1. We calculated the pairwise cosine distance between each of the *de novo* signature mutational profiles.
2. We performed hierarchical clustering and used the base R function cutree() to group signature profiles over the range of all possible cluster sizes (min no. clusters = 2; max no. of clusters = number of signature profiles for the respective mutation type).
3. We next calculated the silhouette score at each cluster size to determine the optimum number of clusters.
4. Finally, we grouped the signature profiles according to the optimum number of clusters. This yielded in total 45 de novo signature clusters (see Supp. Data 4).

For certain *de novo* signature clusters we were able to manually assign the potential etiology by relying on the average similarity to the COSMIC reference mutational signatures. For instance, SBS_denovo_clust_3 represented a collection of *de novo* signatures highly similar to the reference SBS2 and SBS13 from COSMIC, linked to APOBEC mutagenesis. In many cases the mutational signatures displayed an aggregation of both mutational spectra (SBS+SBS13) preventing the reference annotation in the first step of our pipeline. Similarly, DBS_denovo_clust_3 and DBS_denovo_clust_6 represented a collection of *de novo* signatures similar to the DBS5 of COSMIC, which had been linked to platinum treatment exposure. These *de novo* mutational signatures presented the characteristic CT>[AA or AC] peak of DBS5 COSMIC signature in combination with residual contribution from other DBS channels. Finally, we assigned MMR deficiency as the etiology for several clusters (e.g., ID_denovo_clust_1, see Supp. Data 4) as these clusters were enriched in MMR deficient samples.

Next, for each cancer type, we used a logistic regression to quantify the association between the number of somatic point mutations, indels or double base substitutions (DBS) attributed to a certain mutational signature and the presence/absence of a GIE event. We only considered mutational signatures with known/suspected etiology or with high similarity to a reference COSMIC signature (cosine similarity >=0.85) as well as high incidence in a particular cancer type (i.e., at least 15 samples with a mutational signature exposure greater or equal than 100 SBS, 50 IDs or 25 DBS). 49 mutational signatures fulfilled these filters in at least one cancer type. Moreover, to diminish associations that could be mainly attributed to an elevated molecular age, we also included the exposure to aging mutational signature(s) as an independent variable (SBS1 + SBS5 cumulative exposure as a proxy for molecular age). Independent variables were z-scored. The *Logit()* function (with default parameters) from the statsmodels library was used to perform the logistic regression. This function provides the odds ratio with confidence intervals alongside the p-value of significance for both dependent variables. The p-values were adjusted with a multiple-testing correction using the Benjamini–Hochberg procedure (alpha=0.05).

MMR and HR deficiency

We also tested whether mismatch repair deficiency (MMRd) and Homologous repair deficiency (HRd) were predictive of GIE. The Hartwig analytical pipeline provides the MMRd status of each processed tumor sample (i.e., microsatellite stable or microsatellite unstable). Analogously, the CHORD[14] software was used to evaluate HRd in tumor samples. Fisher's exact test was used to evaluate the significance. A minimum of 5 DNA repair-deficient tumor samples were required to assess the significance. P-values were adjusted with a multiple-testing correction using the Benjamini–Hochberg procedure (alpha=0.05).

DNA viral insertion and Whole Genome Duplication

The presence of viral DNA and whole-genome duplication (WGD) is provided by the Hartwig analytical pipeline. A Fisher's exact test was used to evaluate the significance. A minimum of 5 tumor samples harboring viral DNA insertions were required to assess the significance. P-values were adjusted with a multiple-testing correction using the Benjamini–Hochberg procedure (alpha=0.05).

Immune infiltration deconvolution

For samples with available tumor RNA-Seq data we performed an immune infiltration deconvolution based on the normalized TPM and RPKM values in Hartwig and PCAWG, respectively. More specifically, we implemented 6 different markers of immune infiltration: the natural killer cells (NK) quantification by Patrick Danaher et al.[15], the global immune infiltration, $CD8^+$ T-cells and $CD4^+$ T-cells implemented by Teresa Davoli et al.[16], the T-cell infiltration used by Catherine Grasso et al.[17] and the preliminary IFN-γ profile reported by Mark Ayers et al[18].

Next, we used univariate logistic regression to quantify the association of these measurements with GIE prevalence. Independent variables were z-scored. The *Logit()* function (with default parameters) from the statsmodels library was used to perform the logistic regression. This function provides the odds ratio with confidence intervals alongside the p-value of significance for both dependent variables. The p-values were adjusted with a multiple-testing correction using the Benjamini–Hochberg procedure (alpha=0.05).

HLA-I supertypes

We performed a cancer-type specific Fisher's exact test to assess enrichment of *HLA-I* supertypes with the GIE frequency. Only *HLA-I* supertypes present in at least 50 patients were evaluated. *HLA-I* supertypes were gathered from ref.[19] and manually curated. P-values were adjusted with a multiple-testing correction using the Benjamini–Hochberg procedure (alpha=0.05).

HLA-I divergence

We calculate the germline average and cumulative *HLA-I* divergence[20] as the mean and sum of LILAC's *HLA-I* alleles pairwise divergence. Both measurements were independently regressed against the GIE prevalence in a cancer type specific manner. Following the same methodology used with other features, a logistic regression was used to evaluate the significance of the association.

Pre-biopsy treatment exposure

We also tested whether exposure to pre-biopsy treatment had a predictive value for GIE prevalence. For this analysis, we only relied on metastatic pre-treated samples with available pre-treatment information (N=2,212). Five treatment groups were tested: chemotherapy, radiotherapy, immunotherapy, targeted therapy and hormone therapy because of the prevalence across cancer types. A minimum of 5 treated samples were required to carry out the association between a treatment and GIE in a particular tumor type. Fisher's exact test was used to evaluate the significance. P-values were adjusted with a multiple-testing correction using the Benjamini–Hochberg procedure (alpha=0.05).

Driver alterations

We evaluated whether driver alterations (including mutations, copy number gain and losses) showed a significant positive or negative association with GIE. We defined driver alterations per sample as those reported by Linx (v1.17) with a driver likelihood greater than 0.5. Only cancer types with at least 15 tumor samples were considered. Genes associated with GIE were not considered. Similarly, genes close in the proximity of *SETDB1* and *CD274* (same cytogenetic band) were not considered due to high likelihood of co-amplification. A minimum of 5 mutated samples were required to perform the assessment of association. P-values were adjusted with a multiple-testing correction using the Benjamini–Hochberg procedure (alpha=0.05).

References

1. Robinson, J. *et al.* IPD-IMGT/HLA Database. *Nucleic Acids Res.* **48**, D948–D955 (2020).
2. Shukla, S. A. *et al.* Comprehensive analysis of cancer-associated somatic mutations in class I HLA genes. *Nat. Biotechnol.* **33**, 1152–1158 (2015).
3. Xie, C. *et al.* Fast and accurate HLA typing from short-read next-generation sequence data with xHLA. *Proc. Natl. Acad. Sci. U. S. A.* **114**, 8059–8064 (2017).
4. Lee, H. & Kingsford, C. Kourami: graph-guided assembly for novel human leukocyte antigen allele discovery. *Genome Biol.* **19**, 16 (2018).
5. McGranahan, N. *et al.* Allele-Specific HLA Loss and Immune Escape in Lung Cancer Evolution. *Cell* **171**, 1259-1271.e11 (2017).
6. Lakatos, E. *et al.* Evolutionary dynamics of neoantigens in growing tumors. *Nat. Genet.* **52**, 1057–1066 (2020).
7. Jurtz, V. *et al.* NetMHCpan-4.0: Improved Peptide-MHC Class I Interaction Predictions Integrating Eluted Ligand and Peptide Binding Affinity Data. *J. Immunol. Baltim. Md 1950* **199**, 3360–3368 (2017).
8. Vita, R. *et al.* The Immune Epitope Database (IEDB): 2018 update. *Nucleic Acids Res.* **47**, D339–D343 (2019).
9. Pyke, R. M. *et al.* Precision Neoantigen Discovery Using Large-scale Immunopeptidomes and Composite Modeling of MHC Peptide Presentation. *Mol. Cell. Proteomics MCP* **20**, 100111 (2021).
10. Sarkizova, S. *et al.* A large peptidome dataset improves HLA class I epitope prediction across most of the human population. *Nat. Biotechnol.* **38**, 199–209 (2020).
11. O’Donnell, T. J., Rubinsteyn, A. & Laserson, U. MHCflurry 2.0: Improved Pan-Allele Prediction of MHC Class I-Presented Peptides by Incorporating Antigen Processing. *Cell Syst.* **11**, 42-48.e7 (2020).
12. Seabold, S. & Perktold, J. Statsmodels: Econometric and Statistical Modeling with Python. in 92–96 (2010). doi:10.25080/Majora-92bf1922-011.
13. Tate, J. G. *et al.* COSMIC: the Catalogue Of Somatic Mutations In Cancer. *Nucleic Acids*

Res. **47**, D941–D947 (2019).

14. Nguyen, L., W. M. Martens, J., Van Hoeck, A. & Cuppen, E. Pan-cancer landscape of homologous recombination deficiency. *Nat. Commun.* **11**, 5584 (2020).
15. Danaher, P. *et al.* Gene expression markers of Tumor Infiltrating Leukocytes. *J. Immunother. Cancer* **5**, 18 (2017).
16. Davoli, T., Uno, H., Wooten, E. C. & Elledge, S. J. Tumor aneuploidy correlates with markers of immune evasion and with reduced response to immunotherapy. *Science* **355**, eaaf8399 (2017).
17. Grasso, C. S. *et al.* Genetic Mechanisms of Immune Evasion in Colorectal Cancer. *Cancer Discov.* **8**, 730–749 (2018).
18. Ayers, M. *et al.* IFN-γ–related mRNA profile predicts clinical response to PD-1 blockade. *J. Clin. Invest.* **127**, 2930–2940 (2017).
19. Sidney, J., Peters, B., Frahm, N., Brander, C. & Sette, A. HLA class I supertypes: a revised and updated classification. *BMC Immunol.* **9**, 1 (2008).
20. Pierini, F. & Lenz, T. L. Divergent Allele Advantage at Human MHC Genes: Signatures of Past and Ongoing Selection. *Mol. Biol. Evol.* **35**, 2145–2158 (2018).
